# Aerobic, Resistance, and Combination Training on Health-Related Quality of Life: The STRRIDE-AT/RT Randomized Trial

**DOI:** 10.3389/fspor.2020.620300

**Published:** 2021-02-11

**Authors:** Katherine A. Collins, Liezl B. Fos, Leanna M. Ross, Cris A. Slentz, Paul G. Davis, Leslie H. Willis, Lucy W. Piner, Lori A. Bateman, Joseph A. Houmard, William E. Kraus

**Affiliations:** ^1^Duke University Medical Center, Duke Molecular Physiology Institute, Durham, NC, United States; ^2^Division of Cardiology, Duke University School of Medicine, Durham, NC, United States; ^3^Department of Kinesiology, University of North Carolina at Greensboro, Greensboro, NC, United States; ^4^Genomics and Informatics Center, Collaborative Studies Coordinating Center, University of North Carolina at Chapel Hill, Chapel Hill, NC, United States; ^5^Department of Exercise and Sports Science and Human Performance Laboratory, East Carolina University, Greenville, NC, United States

**Keywords:** exercise training, self-perception, behavior change, overweight, obese, physical activity, quality of life

## Abstract

**Purpose:** The main purpose of this study was to determine the differential effects of aerobic training (AT), resistance training (RT), and a combination of aerobic and resistance training (AT/RT) on changes in self-rated HrQoL measures, including the Short-Form 36 (SF-36) survey and Satisfaction with Physical Function and Appearance survey. We also sought to determine if combination training (AT/RT) has a more or less additive effect compared to AT or RT alone on self-rated HrQoL measures.

**Materials and Methods:** Participants (*n* = 137) completed one of three 8-month exercise interventions: (1) AT: 14 kcal exercise expenditure per kg of body weight per week (KKW; equivalent to roughly 12 miles/week) at 65–80% of peak oxygen consumption; (2) RT: 3 days per week, 8 exercises, 3 sets per exercise, 8–12 repetitions per set; (3) AT/RT: full combination of the AT and RT interventions. The SF-36 survey, Satisfaction with Physical Function and Appearance survey, physical fitness, and anthropometrics were assessed at baseline and post-intervention. Paired *t*-tests determined significant pre- vs. post-intervention scores within groups (*p* < 0.05). Analyses of covariance determined differences in change scores among groups (*p* < 0.05).

**Results:** On average, participants were 49.0 ± 10.6 years old, obese (BMI: 30.6 ± 3.2 kg/m^2^), female (57.7%), and Caucasian (84.7%). Following the 8-month intervention, exercise groups improved peak VO_2_ (all groups), strength (RT and AT/RT), and anthropometric measures (AT and AT/RT). For the SF-36, RT (*p* = 0.03) and AT/RT (*p* < 0.001) significantly improved their physical component score; only AT/RT (*p* < 0.001) significantly improved their mental component score. Notably, all groups significantly improved both their satisfaction with physical function and appearance scores (All Groups: *p* < 0.001 for both outcomes).

**Conclusions:** We found that aerobic, resistance, or combination exercise training improves several components of self-rated HrQoL, including physical function, appearance, and mental well-being.

**Clinical Trial Registration**: No. NCT00275145.

## Introduction

Health-related Quality of Life (HrQoL) is a multi-dimensional concept encompassing physical, emotional, and mental well-being. Poor HrQoL is associated with a decreased ability to perform activities of daily living, presence of physical limitations due to pain, loss of energy, limited ability in social activities, and increased depression or nervousness (Posthouwer et al., 2005). As an individual ages, risk for chronic conditions, disability, and comorbidities tend to rise, resulting in reduced HrQoL (White et al., 2009). Further, from adolescence to older adulthood, negative self-perception has damaging effects on HrQoL (Jackson et al., 2014; Manaf et al., 2016). Self-perception—which includes satisfaction with both physical function (SPF) and physical appearance (SPA)—is an influential component of HrQoL (Reboussin et al., 2000; Awick et al., 2015). Poor SPF is associated with low self-efficacy, greater impairment in functional mobility, less fitness, and depressive symptoms (Katula et al., 2004). Poor SPA is associated with low self-esteem, anxiety, depression, and plays a role in eating disorder etiology (Cook and Harman, 2008; Jackson et al., 2014; Seppälä et al., 2014). Consequently, low self-perception of physical function and appearance can lead to a decline in overall HrQoL (Homan and Tylka, 2014; Seppälä et al., 2014; Wang et al., 2018).

Exercise is an effective means for improving HrQoL and body image (Campbell and Hausenblas, 2009). Participation in exercise reduces weight gain, lowers risk for falls, improves physical function, reduces feelings of anxiety and depression, and reduces risk for cardiovascular disease, hypertension, type 2 diabetes, adverse blood lipid profile, and certain cancers (Physical Activity Guidelines Advisory Committee, 2008, 2018). Further, participation in both aerobic and resistance training has been shown to be associated with decreased all-cause mortality (Stamatakis et al., 2017). The 2018 U.S. Physical Activity Guidelines Advisory Committee (Physical Activity Guidelines Advisory Committee, 2018) reported physically active adults were more likely to report better HrQoL compared to sedentary adults (Physical Activity Guidelines Advisory Committee, 2018; DiPietro et al., 2019; Erickson et al., 2019). However, information on the relative effects of different exercise training modes–resistance training, aerobic training or a combination thereof—on self-rated HrQoL is lacking. Further, the U.S. Physical Activity Guidelines call for both aerobic and resistance training modes to be performed for greatest health improvements; however, little literature has examined the impact of combination training on HrQoL (DiPietro et al., 2019; Erickson et al., 2019). The second Studies of a Targeted Risk Reduction Intervention through Defined Exercise randomized trial – STRRIDE AT/RT – investigated the independent and combined effects of aerobic training (AT) and resistance training (RT) on health outcomes. Thus, as a secondary analysis from STRRIDE AT/RT, the main purpose of this study was to determine the differential effects of AT, RT, and a combination of aerobic and resistance training (AT/RT) on changes in self-rated HrQoL measures, including the Short-Form 36 survey and Satisfaction with Physical Function and Appearance survey. This secondary analysis of a prospective exercise training intervention also offered the opportunity to explore whether there is a more or less additive effect of combination training on self-rated HrQoL among overweight or obese adults at risk for cardiometabolic disease.

## Materials and Methods

### Study Design

In the STRRIDE-AT/RT randomized trial (NCT00275145; conducted from 2004–2008), participants completed physical fitness, body composition, and HrQoL assessments prior to and following the end of an 8-month supervised exercise intervention. Participants were recruited continuously between 2004 and 2008. Participants were randomized to either AT, RT, or AT/RT; there was no control group included in this randomized trial. Randomization was performed with a standard computer-based random number generator using a randomized design, blocked by gender, race, and study site. The STRRIDE AT/RT study protocol was approved by the institutional review boards at Duke University (Duke) and ECU.

### Participants

Potential participants (*n* = 3,145) responded to local advertisements and were screened by phone. Of these, 234 met inclusion criteria and were recruited into the study, 75% were recruited at Duke and the remaining 25% at ECU. Inclusion criteria were as follows: age 18–70 years, sedentary (dedicated leisure time physical activity <1 day per week), body mass index (BMI) 26–35 kg/m^2^, and mild to moderate dyslipidemia (low density lipoprotein cholesterol 130–190 mg/dL and/or high density lipoprotein cholesterol <40 mg/dL for men or <45 mg/dL for women). Participants were non-smokers without a history of diabetes, hypertension, or coronary artery disease. The use of statin drugs was an exclusion criterion. After informed, written consent, participants were asked to maintain their current lifestyle during a 4-month run-in period, followed by randomization into one of three exercise training groups. The purpose of the run-in period was to discourage individuals who were not serious about the study commitment and thus reduce the dropout rate that may occur after randomization. Participants were compensated for participation in the study. Of the 234 recruited, 38 participants dropped out during the run-in period, leaving 196 participants for randomization. Of the participants who were randomized, 73.5% (*n* = 144) completed the trial, 133 participants completed the Short-Form 36 (SF-36) survey, and 126 completed the Satisfaction with Physical Function and Appearance Survey. Demographic data were collected prior to the 4-month run-in period. All other measures in this analysis – HrQoL surveys, anthropometrics, and physical fitness – were assessed at baseline (pre-intervention) and post-intervention (16–24 h following the final exercise bout).

### Exercise Training

The exercise groups were as follows: (1) AT: 14 kcal exercise expenditure per kg of body weight per week (KKW; equivalent to roughly 12 miles/week) at 65–80% of peak oxygen consumption; (2) RT: 3 days per week, 8 exercises, 3 sets per exercise, 8–12 repetitions per set; (3) AT/RT: full combination of the AT and RT interventions (Bateman et al., 2011).

#### Aerobic Training

For participants in the AT and AT/RT groups, a ramp period of 8–10 weeks was designed to gradually increase the amount of aerobic exercise to the prescribed amount. Once achieved, the prescribed amount of aerobic exercise was maintained for the remainder of the 8-month training period. Details about the prescribed and actual exercise training amounts, intensity, and frequency are provided in Table 1. The aerobic exercise modalities included treadmill, elliptical trainers, cycle ergometers, or a combination. All aerobic exercise sessions were verified either by direct supervision from fitness staff and/or use of a heart rate monitor that provided recorded, downloadable data (Polar Electro, Inc; Woodbury, NY). Intensity of the AT program was based on and maintained by using heart rate zones; therefore, participants in AT/RT performed the AT exercise first, followed by RT. The total amount of aerobic exercise minutes was determined with a cardiorespiratory fitness test, as all participants were prescribed a specific amount of exercise per unit body weight [i.e., KKW]. Individuals with greater fitness required less time to expend the prescribed number of calories per week. Exercise frequency was not prescribed for aerobic training; however, participants were encouraged not to exceed 60 min/day. Participants were encouraged to exercise at least three times per week. Aerobic compliance percentages were calculated each week as a percentage, equal to the number of minutes completed within the prescribed heart rate range, divided by the number of total weekly minutes prescribed.

**Table 1:** Exercise prescription and adherence by group.

	**Aerobic training (*n* = 44)**	**Resistance training (*n* = 48)**	**Aerobic + Resistance training (*n* = 45)**
**Resistance Rx**			
Intensity	-	Progressive	Progressive
Rx amount, sets/wk	-	72	72
Adherence %	-	83.0 (12.8)	81.4 (14.0)
Average frequency		2.5	2.5
**Aerobic Rx**			
Intensity, % peak VO_2_	65–80%	-	65–80%
Prescription amount			
kcal/kg/wk	14	-	14
miles/wk	12		12
min/wk	131.2 (24.8)	-	133.4 (25.0)
Adherence %	89.7 (10.0)	-	82.2 (17.2)
Average frequency	3.0		2.8

*Values are means (SD). Rx = exercise prescription for AT and RT groups. Rx amount = (kcal/kg/wk) = kcal exercise expenditure per kg of body weight per week. Average frequency refers to the group average number of sessions attended per week*.

#### Resistance Training

For participants randomized to RT or AT/RT, the ramp period began with one set during *weeks 1–2*, two sets during *weeks 3–4*, and built up to the prescribed three set amount on *week 5*, which was maintained throughout the remainder of the 8-month training period. The groups were prescribed three sessions per week (on non-consecutive days), three sets each of 8–12 repetitions of upper body (bench press, military (or overhead) press, lat pull, seated row, back extension (or bicep flexion and triceps extension), and lower body (leg extension, leg flexion, and leg press) exercises. Throughout the intervention, the amount of weight lifted was increased by 5 pounds each the time participant performed 12 repetitions with proper form on all three sets on two consecutive workout sessions to ensure a progressive RT stimulus.

All training sessions at Duke were verified by direct supervision of fitness staff and/or the FitLinxx Strength Training Partner (FitLinxx; Norwalk, CT). Throughout each workout, the “training partner” captured and stored information including total amount of weight lifted, verified by infrared laser, and the number of repetitions and sets completed within the pre-programed speed and range of motion limits. At the ECU site, sessions were confirmed via direct supervision by fitness staff. To accommodate those individuals who were randomized to AT/RT, aerobic and resistance training sessions were combined into one session to obviate the need to make twice the number of visits to the center.

### Short Form−36 Survey

The SF-36 survey was used to measure self-perceived physical and mental health over that past 4 weeks before and following the intervention period. This is a 36-item survey scored into eight domains: (1) physical functioning, (2) role-physical, (3) bodily pain, (4) general health, (5) vitality, (6) social functioning, (7) role-emotional, and (8) mental health. Physical Component Score is comprised of the following 4 domains: physical functioning, role-physical, bodily pain, and general health. Mental Component Score is measured with the following 4 domains: role-emotional, social functioning, vitality, and mental health. The validity and reliability of the SF-36 have been established, and there are standardized norms available for comparative purposes (Ware and Sherbourne, 1992; Ware et al., 2000). Participants' raw scores were converted into scale scores ranging from 0 to 100, with higher scores representing better HrQoL or higher functioning for all scales (Ware et al., 1996). The SF-36 was scored by blinded assessors.

### Satisfaction With Physical Function and Appearance Survey

The Satisfaction with Physical Function and Appearance survey was used to measure participant-perceived satisfaction with physical function and appearance before and following the intervention period. This survey was developed by Ray et al. and has been validated in several randomized controlled trials to pinpoint the satisfaction with HrQoL and physical activity participation (Ray et al., 1996; Reboussin et al., 2000). This nine-question survey contained five questions regarding physical function and four questions on physical appearance. Participants answered the following questions regarding physical function: “Over the past 4 weeks, how satisfied have you been with (1) your overall level of physical fitness? (2) the muscle strength in your legs? (3) your level of endurance or stamina? (5) your overall level of energy? (6) your physical ability to do what you want or need to do?” The following questions were asked regarding physical appearance: “Over the past 4 weeks, how satisfied have you been with (4) your muscle tone? (7) your weight? (8) your shape? (9) your overall physical appearance?” Each item was rated on a 7-point Likert scale ranging from −3 to 3 with the following terms: (−3) very dissatisfied, (−2) somewhat dissatisfied, (−1) a little dissatisfied, (0) neither, (+1) a little satisfied, (+2) somewhat satisfied, and (+3) very satisfied. Questions 1, 2, 3, 5, and 6 were averaged together to generate SPF score. Similarly, questions 4, 7, 8, and 9 were averaged together to generate SPA score. Greater scores indicate greater satisfaction with physical function and/or appearance. The Satisfaction with Physical Function and Appearance survey was scored by blinded assessors.

### Anthropometric Measures

All anthropometric measurements were performed by trained study staff. At the Duke University clinical site, body composition was assessed using the BOD POD air displacement plethysmography method (Life Measurement, Concord, CA) on all participants before and following the intervention period. At ECU, body composition was assessed using dual-energy x-ray absorptiometry (DXA). As previously reported, measurements with BOD POD and DXA are highly correlated (0.94) with one another (Ball and Altena, 2004). Further, with the focus of this analysis being pre- to post-intervention change scores, any differences between the study sites due to techniques used to assess body composition would not affect the data interpretation. Height and body weight were assessed with the participant in light weight clothing and shoes removed. Body weight was assessed using a calibrated digital scale to the nearest 0.1 kg, with the average of three weights taken over 2 weeks, on different days, being used for each time point. Body weight was measured three times (before each intravenous glucose tolerance test, cardiopulmonary exercise test, and body composition testing), therefore we averaged these together to produce the most stable weight outcome. Height was assessed using a stadiometer to the nearest 0.25 cm, measured one time. Body mass index (BMI) was calculated from height and weight measurements. Waist circumference was measured at the minimal waist (the lowest circumference measurement above the umbilicus and below the xiphoid). Our laboratory has previously shown the minimal waist measure to be highly correlated to metabolic health, compared to the umbilicus waist measurement (Willis et al., 2007).

### Physical Fitness Measures

All physical fitness measures were performed by trained study staff. Peak VO_2_ was determined via maximal cardiopulmonary exercise tests with a 12-lead ECG and expired gas analysis on a treadmill using a TrueMax 2400 Metabolic Cart (ParvoMedics, Sandy, UT) before and after the exercise intervention, as described previously (Duscha et al., 2005). Exercise tests were performed under medical supervision and were conducted by exercise physiologists. The two highest, consecutive, 15 second readings from each test were averages to determine absolute peak VO_2_ (L/min). In RT and AT/RT participants, the upper and lower body total amounts of weight lifted (TWL) from a single session during week 5 were used as the baseline measure of overall strength. The same measurements from a single session at week 32 were used as the end of training measure of overall strength. The difference in these two amounts constituted the overall strength gains expressed in pounds lifted/session. TWL was recorded each week either by a supervising exercise professional at East Carolina University site or electronically by the FitLinxx Strength Training Partner system (FitLinxx, Norwalk, CT) at the Duke University site.

### Statistical Analyses

Data in this secondary analysis were analyzed using Statview (SAS Institute, Cary, NC) or JMP (SAS Institute, Cary, NC). Two-tailed, paired *t*-tests were used to determine whether the pre- vs. post-intervention score for changes within each group were significant (**Table 3**). A *p-*value of <0.05 was considered significant. To determine between group differences, analysis of covariance (ANCOVA), with baseline values used as a covariate was conducted. When the analysis was impressionable (*p* < 0.10), Tukey-Kramer *post-hoc* analysis was performed to determine the differences between the groups (**Table 4**, **Figures 2**, **3**). *P*-values <0.05 were considered significant in *post-hoc* testing. Prior to data analysis, assumptions for conducting a paired *t*-test and ANCOVA were assessed, with data meeting all necessary assumptions. There were no a priori power calculations because the variables in the present article were not primary outcome variables for the STRRIDE AT/RT study. The analyses present were performed “per protocol.”

## Results

**Figure 1:**
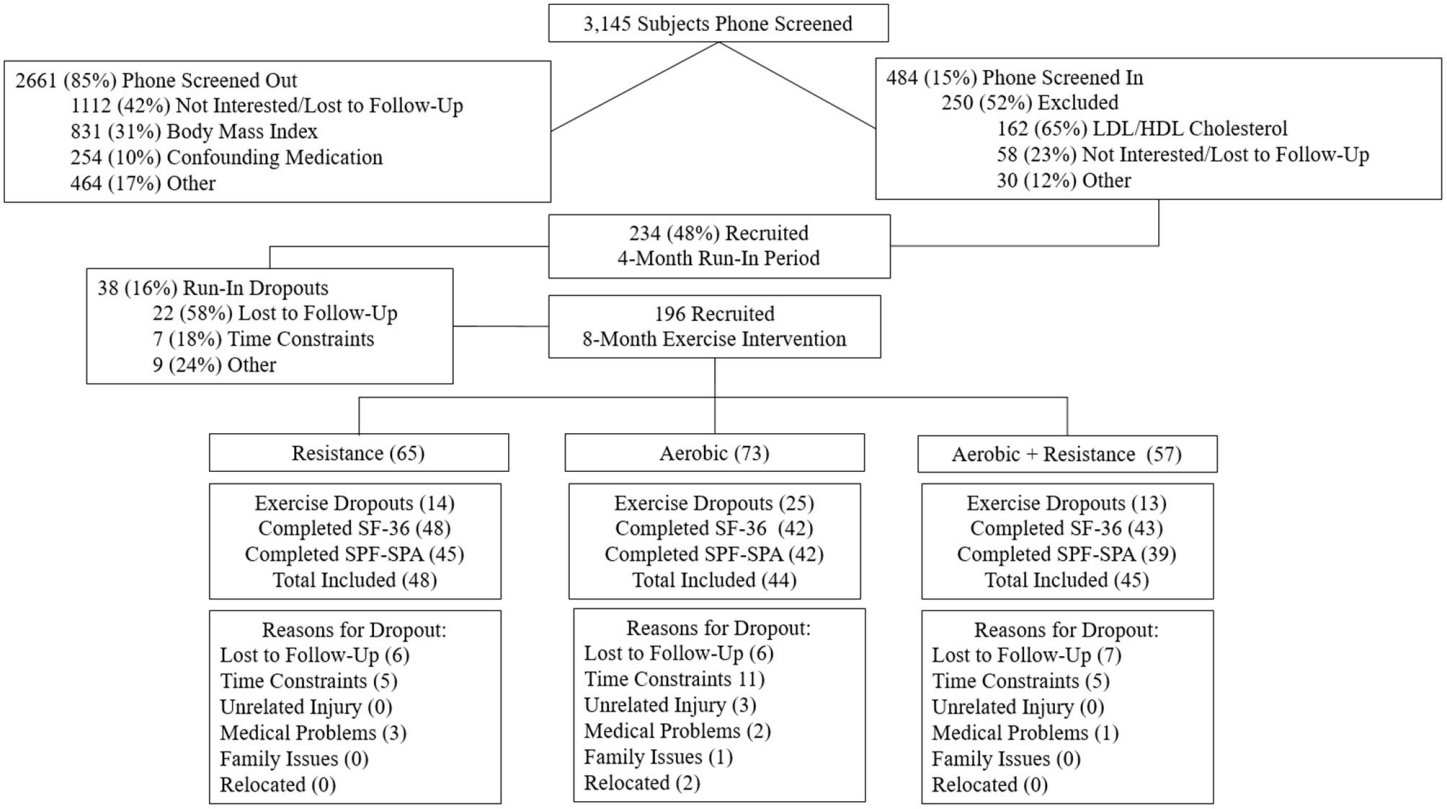
Flowchart of screening and randomization inclusion and exclusion.

Of the 196 participants randomized, 137 participants had data for either the SF-36 (*n* = 133) or Satisfaction with Physical Function and Appearance (*n* = 126) surveys. Figure 1 describes the flow of participants from recruitment to post-intervention testing. The baseline demographics are presented for each group in Table 2. Participants were on average 49.0 ± 10.6 years old, obese (BMI: 30.6 ± 3.2 kg/m^2^), female (57.7%), and Caucasian (84.7%). The exercise prescription and adherence are described in Table 1. Participants in AT were more adherent to the prescribed aerobic exercise intervention compared to participants in the AT/RT (89.7 vs. 82.2%, respectively; *p* = 0.016 for difference among groups). The main HrQoL outcomes of physical component score, mental component score, SPF, and SPA were not significantly different at baseline among groups.

**Table 2:** Baseline characteristics by exercise groups.

	**Aerobic training (*n* = 44)**	**Resistance training (*n* = 48)**	**Aerobic + Resistance training (*n* = 45)**
Age, yr	50.1 (9.9)	50.2 (11.6)	46.8 (9.9)
Sex, % Female	54.5	60.4	57.8
Race, %			
Caucasian	86.4	83.3	84.5
African American	13.6	14.6	13.3
Asian	-	2.1	-
Indian	-	-	2.2
Body weight, kg	88.5 (10.5)	88.5 (15.7)	90.4 (12.1)
Body mass index, kg/m^2^	30.6 (3.0)	30.6 (3.4)	30.7 (3.4)

*Values are means (SD)*.

Baseline and change scores for SF-36 individual domains, physical component score, mental component score, SPF, SPA, physical fitness, and anthropometric variables are presented for each group in Table 3. Following the 8-month intervention, all groups significantly improved peak VO_2_, ranging from 1.1 ± 2.3 to 4.0 ± 3.2 mL/kg/min (*p* < 0.001 for all groups). Both RT and AT/RT significantly improved the TWL from baseline by 9073.0 ± 5561.2 and 7699.3 ± 5521.7 lb., respectively (*p* < 0.001 for both groups). For anthropometric variables, both AT and AT/RT reduced their minimal waist circumference by −1.0 ± 2.7 and −2.1 ± 2.9 cm, respectively (*p* < 0.05 for both groups). Only AT/RT experienced reductions in fat mass (−2.3 ± 3.1 kg; *p* < 0.001) and hip circumference (−2.1 ± 2.6 cm; *p* < 0.001). Differences among groups are presented in Table 4, with Tukey's *post-hoc* analysis performed only when there was an impressionable *p*-value.

**Table 3:** Baseline and change values for satisfaction with physical function and appearance, SF-36, anthropometric, and fitness variables.

		**Aerobic training**		**Resistance training**		**Aerobic** **+** **Resistance training**	
		**Baseline^α^**	**Δ**	**Baseline^α^**	**Δ**	**Baseline^α^**	**Δ**
**Satisfaction with**		*n* = 42		*n* = 45		*n* = 39	
	Physical function	−0.4 (1.6)	2.1 (1.6)^**^	−0.5 (1.6)	2.0 (1.9)^**^	−0.7 (1.7)	2.6 (2.0)^**^
	Physical appearance	−1.6 (1.3)	1.3 (1.3)^**^	−1.5 (1.7)	1.3 (1.7)^**^	−1.2 (1.8)	1.8 (2.3)^**^
**SF-36**		*n* = 42		*n* = 48		*n* = 43	
	Physical component score	76.5 (5.1)	**1.6 (5.7)**^†^	76.1 (6.8)	1.7 (5.2)^*^	75.4 (6.2)	3.8 (5.4)^**^
	Physical functioning	78.2 (10.3)	4.3 (9.9)^*^	78.9 (10.2)	3.4 (7.7)^*^	76.0 (20.9)	2.9 (11.5)^**^
	Role-physical	90.2 (14.1)	3.6 (16.9)	85.4 (21.4)	3.8 (19.8)	90.3 (13.6)	2.3 (14.4)
	Bodily pain	81.6 (19.4)	−1.9 (25.1)	82.3 (18.5)	−2.7 (17.5)	74.0 (16.1)	**4.7 (16.1)**^†^
	General health	63.3 (14.1)	3.4 (14.6)	63.1 (23.1)	**5.5 (19.2)**^†^	63.3 (20.0)	10.6 (19.3)^**^
	Mental component score	58.5 (5.5)	−0.1 (6.6)	57.6 (7.4)	1.0 (6.5)	56.8 (8.0)	3.3 (5.9)^**^
	Vitality	57.9 (16.5)	4.7 (13.4)^*^	60.3 (18.0)	**3.9 (15.7)**^†^	56.6 (20.4)	9.7 (18.7)^*^
	Social functioning	82.0 (11.9)	−0.3 (12.3)	79.4 (12.7)	−0.3 (17.0)	78.2 (16.8)	1.7 (12.4)
	Role-emotional	92.0 (13.3)	0.5 (15.8)	86.0 (20.0)	0.2 (20.6)	85.8 (19.5)	6.7 (16.7)^*^
	Mental health	75.0 (13.9)	−1.1 (12.5)	72.3 (20.1)	2.7 (17.5)	71.8 (19.7)	6.1 (15.8)^*^
**Anthropometric and fitness**		*n* = 44		*n* = 48		*n* = 45	
	Fat mass (kg)	33.9 (8.1)	−0.7 (3.8)	34.3 (8.8)	0.1 (2.6)	34.9 (8.9)	−2.3 (3.1)^**^
	Minimal waist circumference (cm)	97.0 (9.5)	−1.0 (2.7)^*^	95.4 (9.6)	0.01 (2.0)	96.8 (10.0)	−2.1 (2.9)^**^
	Hip circumference (cm)	111.0 (6.5)	−0.6 (2.8)	112.0 (8.4)	0.03 (2.9)	113.6 (8.9)	−2.1 (2.6)^**^
	Total weight lifted (lbs)	-	-	19273.4 (7954.8)	9073.0 (5561.2)^**^	18906.7 (5873.8)	7699.3 (5521.7)^**^
	Peak VO_2_ (ml/kg/min)	28.0 (6.0)	3.4 (3.5)^**^	26.0 (6.0)	1.1 (2.3)^**^	27.2 (5.9)	4.0 (3.2)^**^

*Values are means (SD). Δ represents the change from baseline to post-intervention*.

α *A higher score indicates a better health state*.

† *p-value < 0.1*;

* *p-value < 0.05*;

** *p-value < 0.001. Bold values indicate significant or trending toward significant values*.

**Table 4:** ANCOVA and *post-hoc* comparison values for SF-36 domains, physical fitness, and anthropometric variables.

	**Model (*F* Ratio)**	**Model (*p*-value)**	**Group effect (*F* Ratio)**	**Group effect (*p*-value)**	***Post-Hoc*** **within group Δ (*****SD*****)**		
					**AT vs. RT**	**AT vs. AT/RT**	**RT vs. AT/RT**
**SF-36 domains**							
Physical functioning	36.92	<0.001^**^	1.88	0.157	-	-	-
Role-physical	30.91	<0.001^**^	0.56	0.573	-	-	-
Bodily pain	1.83	0.146	1.57	0.212	-	-	-
General health	24.47	<0.001^**^	3.14	0.047^*^	3.4 (14.6) vs. 5.5 (19.2)	3.4 (14.6) vs. 10.6 (19.3)^*^	5.5 (19.2) vs. 10.6 (19.3)
Vitality	19.36	<0.001^**^	1.67	0.193	-	-	-
Social functioning	13.15	<0.001^**^	0.21	0.812	-	-	-
Role-emotional	11.86	<0.001^**^	1.83	0.164	-	-	-
Mental health	17.85	<0.001^**^	2.51	**0.085^†^**	−1.1 (12.5) vs. 2.7 (17.5)	–**1.1 (12.5) vs. 6.1 (15.8)^†^**	2.7 (17.5) vs. 6.1 (15.8)
**Physical fitness and anthropometrics**							
Fat mass	7.23	<0.001^**^	6.77	0.002^**^	−0.7 (3.8) vs. 0.1 (2.6)	−0.7 (3.8) vs.−2.3 (3.1)	0.1 (2.6) vs.−2.3 (3.1)^**^
Minimal waist circumference	4.99	0.003^**^	8.34	<0.001^**^	−1.0 (2.7) vs. 0.01 (2.0)	–**1.0 (2.7) vs.−2.1(2.9)^†^**	0.01 (2.0) vs.−2.1(2.9)^**^
Hip circumference	6.56	<0.001^**^	6.12	0.003^**^	−0.6 (2.8) vs. 0.03 (2.9)	–**0.6 (2.8) vs.−2.1 (2.6)^†^**	0.03 (2.9) vs.−2.1 (2.6)^**^
Total weight lifted	15.46	<0.001^**^	1.28	0.262	-	**-**	-
Peak VO_2_	8.14	<0.001^**^	12.15	<0.001^**^	3.4 (3.5) vs. 1.1 (2.3)^**^	3.4 (3.5) vs. 4.0 (3.2)	1.1 (2.3) vs. 4.0 (3.2)^**^

*Tukey's HSD was performed only if the ANCOVA group effect was impressionable (p < 0.1)*.

† *p-value < 0.1*;

* *p-value < 0.05*;

** *p-value < 0.001. Bold values indicate significant or trending toward significant values*.

**Figure 2:**
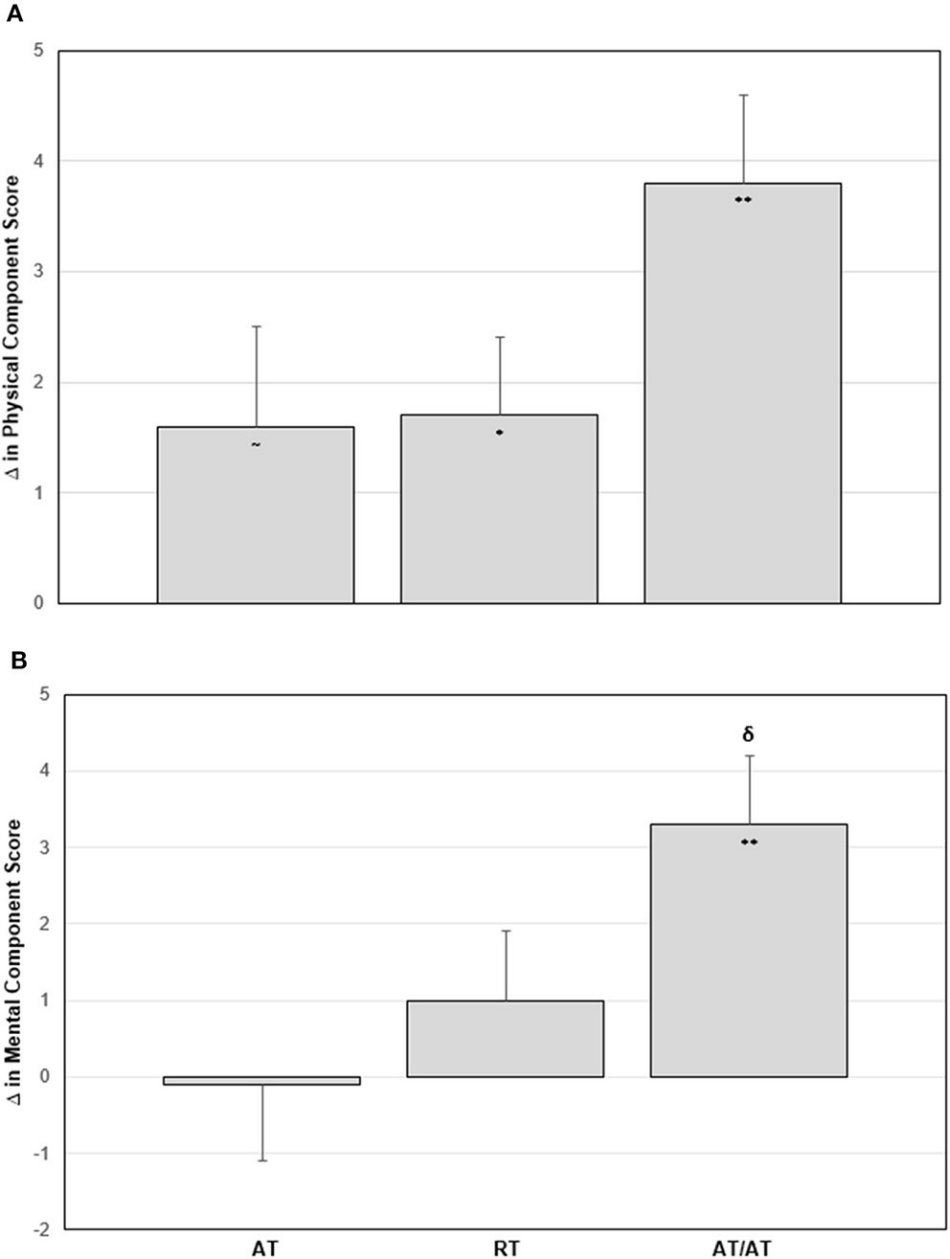
**Panel A**: Change in physical component score by group. **Panel B**: Change in mental component score by group. Error bars indicate SE. Significant within group change: ~*p*-value < 0.1; *p-value < 0.05; **p-value < 0.001. ^δ^*p*-value < 0.1 Tukey's *Post-Hoc* Test compared with aerobic training.

At baseline, all three exercise groups began with average SF-36 scores comparable to the general US population for the physical component score, mental component score, and individual domain scores (McDowell, 2006). Following the intervention period, both RT (*p* = 0.03) and AT/RT (*p* < 0.001) significantly increased their physical component score by 1.7 ± 5.2 and 3.8 ± 5.4 points, respectively (Figure 2, **Panel A**). After adjusting for baseline values, there were no significant differences among groups for change in physical component scores (*F* = 2.10, *p* = 0.127). For change in mental component scores following the intervention, only AT/RT significantly experienced an increased score (3.3 ± 5.9 points; *p* < 0.001; Figure 2, **Panel B**). There was an impressionable *p*-value among groups for change in mental component score (*F* = 2.51, *p* = 0.085*)*. Tukey's HSD *post-hoc* testing revealed only a trending toward significant difference between AT and AT/RT (*p* = 0.08; Figure 2, **Panel B**). When evaluating changes in each of the domain scores, AT/RT significantly improved “physical functioning” (*p* < 0.001), “general health” (*p* < 0.001), “vitality” (*p* = 0.001), “role-emotional” (*p* = 0.012), and “mental health” (*p* = 0.015) domain scores. RT only experienced a significant improvement in “physical functioning” (*p* = 0.003). Finally, AT experienced a significant improvement “physical functioning” (*p* < 0.001) and “vitality” (*p* = 0.03). Table 4 contains significant differences among groups for each SF-36 domain, after adjusting for baseline values.

**Figure 3:**
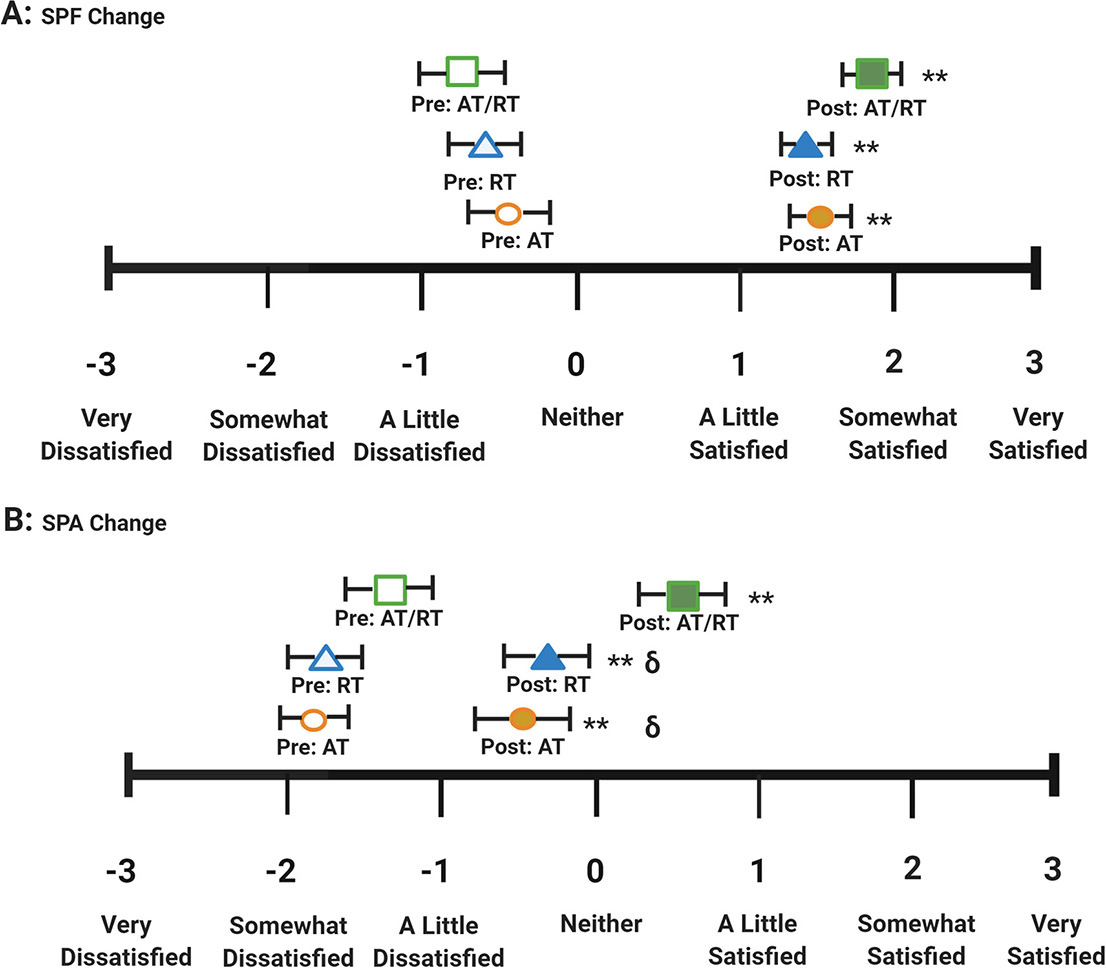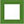 AT/RT pre-intervention score. 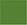 AT/RT post-intervention score. 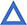 RT pre-intervention score. 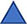 RT post-intervention score. 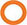 AT pre-intervention score. 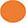 AT post-intervention score. **Panel A**: Satisfaction with physical function pre/post scores by intervention group. **Panel B**: Satisfaction with physical appearance pre/post scores by intervention group. Error bars indicate SE. Significant within group change: **p-value < 0.001. ^δ^*p*-value < 0.01 Tukey's *Post-Hoc* Test compared with aerobic + resistance training.

At baseline, all three groups had overall negative average SPF and SPA scores, indicating overall dissatisfaction with both physical function and physical appearance. Figure 3 displays the average pre- and post-intervention scores for SPF and SPA by group. Following the intervention period, all exercise groups significantly increased both SPF (*p* < 0.001 for all groups; Figure 3, **Panel A**) and SPA (*p* < 0.001 for all groups; Figure 3, **Panel B**) scores. After adjusting for baseline values, there were no significant differences between groups for change in SPF score (*F* = 1.80; *p* = 0.169). There was an impressionable *p*-value among groups for change in SPA score (*F* = 4.78; *p* = 0.010). Tukey's HSD *post-hoc* testing revealed a significant difference between RT and AT/RT (*p* = 0.018; Figure 3, **Panel B**), as well as, between AT and AT/RT (*p* = 0.025; Figure 3, **Panel B**). The changes in SPF score following the intervention resulted in overall positive ratings for each exercise group (AT = 1.7 ± 1.2; RT = 1.5 ± 1.0; AT/RT = 1.9 ± 1.0). However, changes in SPA score following the intervention resulted in a positive rating only for AT/RT (0.7 ± 1.4). AT and RT maintained an overall negative rating of SPA (AT: −0.3 ± 1.4; RT: −0.2 ± 1.4) even though a statistically significant change was found, indicating overall dissatisfaction with appearance following the exercise intervention.

## Discussion

Participation in exercise has been shown to improve measures of HrQoL (Martin et al., 2009; Reid et al., 2010; Pucci et al., 2012). However, little research has been done comparing the effects of AT, RT, and the combination there-of on self-rated HrQoL. The STRRIDE AT/RT study provided a unique opportunity to determine if an 8-month combination AT/RT exercise intervention had a more or less additive effect on measures of self-rated HrQoL compared to AT and RT alone using a secondary analysis. In our study design, AT/RT performed an additive combination of the AT and RT interventions, almost doubling the amount of exercise compared to AT or RT. Therefore, we had the unique opportunity to determine if AT/RT resulted in a more or less additive change in physical component score, mental component score, SPF score, and SPA score compared to AT or RT alone.

The primary findings of the parent STRRIDE-AT/RT study were as follows: (1) the RT program significantly improved peak VO_2_ and strength, with all other cardiometabolic outcomes not effected, (2) the AT program resulted in significant improvements in body mass, peak VO_2_, and triglycerides, and (3) the full combination of AT/RT resulted in significant improvements in body mass, peak VO_2_, strength, triglycerides, waist circumference, diastolic blood pressure, mean arterial blood pressure, ATP III score, and Metabolic Syndrome *z*-score (Bateman et al., 2011). The results of this secondary analysis on changes in anthropometric and physical fitness outcomes mirror those previously published. We found AT/RT exercise resulted in significantly improved objective measures of health—cardiorespiratory fitness, strength, and anthropometrics. RT exercise resulted in significant improvement in measures of strength, AT exercise resulted in significant improvement in anthropometrics, and both significantly improved cardiorespiratory fitness.

In this secondary analysis, we found all exercise groups experienced significant improvements in self-rated HrQoL measures. In the SF-36, AT/RT had the greatest improvement in physical component score, mental component score, and individual domain scores. When comparing the effects of AT and RT alone to AT/RT, we found an additive effect for change in physical component score in AT/RT following the intervention (Figure 2, **Panel A**). Interestingly, we observed a more than additive effect of AT/RT on mental component score (Figure 2, **Panel B**). In the Satisfaction with Physical Function and Appearance survey, we found significant improvements in all exercise interventions for both SPF and SPA scores. When comparing the effects of AT and RT alone to AT/RT, we found there was a less than additive effect observed in AT/RT for both SPF and SPA score compared to AT and RT alone (Table 3); However, change in SPF and SPA in AT/RT resulted in a numerically greater change compared to either AT or RT alone, following the intervention.

Although the SF-36 is a well-known and broadly used questionnaire, depending on the population, the survey can lack the sensitivity needed to detect a significant change in self-rated HrQoL. In the U.S., mean scores for individual domains range from 70.9 to 84.3 on a scale of 0–100 (excluding vitality which has a lower mean of 58.3), with physical component domain scores typically greater than mental component domain scores (McDowell, 2006). Our average baseline scores for physical component domains ranged from 63.1 (“general health”) to 90.3 (“role-physical”); average baseline scores for mental component domains ranged from 56.8 (“vitality”) to 92.0 (“role-emotional”). Though the domain scores in our cohort of previously sedentary adults at risk for cardiometabolic disease were comparable to U.S. general population scores, they were high on a scale from 0 to 100, potentially limiting the ability to detect large changes in self-rated HrQoL (McDowell, 2006). Further, the SF-36 questions around physical function and mental health (e.g., “Does your health now limit you in these activities? Lifting or carrying groceries, climbing one flight of stairs,” etc.) may be too specific or inappropriate to detect a change in our cohort, comprised of highly functioning, relatively healthy adults who performed an intense exercise intervention.

On the other hand, the Satisfaction with Physical Function and Appearance survey utilizes a 7-point Likert scale, conferring more sensitivity to change in our cohort. Therefore, inclusion of the Satisfaction with Physical Function and Appearance survey in this study provided an additional lens by which to evaluate changes in self-rated HrQoL following an exercise intervention. Moreover, using a single Likert scale for all response choices in the Satisfaction with Physical Function and Appearance survey may limit bias from the respondents. Various arrangements of response choices and change of response scales throughout the SF-36 survey may cause bias in how the participants answer each set of questions, therefore diminishing potential sensitivity to the measure (Choi and Pak, 2005). The participants in our study reported overall dissatisfaction with their physical function and appearance at baseline. By the end of the intervention, all three exercise groups experienced significant improvements in both SPF and SPA scores; this illustrates the utility of this survey to assess components of self-rated HrQoL in a population at risk for cardiometabolic disease development.

Few large randomized controlled trials have examined the effects of AT and RT on overweight or obese adults. In the DREW (Dose Response to Exercise in Women) study (Martin et al., 2009), sedentary, post-menopausal women were randomized to either a control group or one of three aerobic exercise interventions. A significant, positive dose-response relationship was found between amount of aerobic exercise performed and improvements in physical and mental QoL components (Martin et al., 2009). Among individuals with type 2 diabetes in the HART-D study, AT, RT, and combined AT/RT exercise training improved physical component score; however, the effects on mental component score were limited, with improvements favoring AT/RT and RT (Myers et al., 2013). Moreover, in our study of sedentary adults at risk for cardiometabolic disease, we found greater changes in the physical component and mental component scores with AT/RT compared with AT or RT alone.

An 8-week AT vs. RT randomized exercise intervention assessing changes in body image among college-aged women with pre-existing body image concerns found both AT and RT significantly improved body image satisfaction scores in three body image measures (Ginis et al., 2005). Further, when comparing change in body image among groups, AT had significantly greater improvements in body image satisfaction scores compared to RT (Ginis et al., 2005). We found a significant group difference between AT and AT/RT for SPF change, and no group differences for SPA change. These differences may be attributed to study design and population. More evidence is needed to determine whether AT, RT, or a combination AT/RT program is the most effective for improving self-perception of appearance and function.

To the best of our knowledge, this is one of the first studies to explore the differential effects of exercise modes separately and in full combination on changes in self-rated HrQoL. Further, the 8-month duration of the intervention resulted in significant changes among objective measures of cardiorespiratory fitness and anthropometrics; therefore, this may be a sufficient duration to see significant changes in self-rated HrQoL. In addition, our study employed two different measures of self-rated HrQoL, allowing us to assess the effects of our exercise interventions on different dimensions of HrQoL.

We recognize the limitations of our study, which includes the recruited population contained men and women motivated to perform exercise in a semi-supervised setting; this may limit the generalizability of our findings to other populations. Using questionnaires to capture perceived HrQoL measures can result in inclusion of false answers, differences in understanding and interpretation of questions, lack of personalization, and unanswered questions. Another limitation is this study was designed as an efficacy study, not an intention to treat study. Further, the data collected in this study were collected between 2004–2008, therefore it is unclear if the outcomes measured here would be the same if the data had been collected more recently today as obesity continues to become visually normalized. Last, this study lacks the ability to detect if the changes seen with AT/RT are due to the addition of RT, or if greater amounts of either AT or RT would have produced a similar effect.

## Conclusion

We found that aerobic and/or resistance exercise training can positively influence self-rated HrQoL measures, with the combination of both modes having the greatest impact. To the best of our knowledge this is one of the first studies to provide evidence that individuals should include both aerobic and resistance training modes in physical activity regiments in order to improve self-rated HrQoL, supporting the U.S. Physical Activity Guidelines.

## Data Availability Statement

The raw data supporting the conclusions of this article will be made available by the authors upon request, without undue reservation.

## Ethics Statement

The studies involving human participants were reviewed and approved by Duke University Institutional Review Board. The patients/participants provided their written informed consent to participate in this study.

## Author Contributions

WK, JH, and CS contributed to the study conception and design. Data collection was performed by LW, LP, LB, and CS. Data analysis and manuscript conception was conducted by KC, LF, LR, PD, and CS. The first draft of the manuscript was written by KC and all authors commented on previous versions of the manuscript. All authors read and approved the final manuscript.

## Conflict of Interest

The authors declare that the research was conducted in the absence of any commercial or financial relationships that could be construed as a potential conflict of interest.
